# Juvenile Spondyloarthritis: focus on uveitis

**DOI:** 10.1186/s12969-020-00463-4

**Published:** 2020-09-10

**Authors:** Achille Marino, Pamela F. Weiss, Timothy G. Brandon, Melissa A. Lerman

**Affiliations:** 1 Department of Pediatrics, Desio Hospital, Via Mazzini 1, Desio, MB Italy; 2 University of Florence, Piazza San Marco 4, Florence, Italy; 3 Department of Pediatrics, Perelman School of Medicine, University of Pennsylvania, 3400 Civic Center Blvd, Philadelphia, PA 19104 USA; 4 Division of Rheumatology, The Children’s Hospital of Philadelphia, 3401 Civic Center Blvd, Philadelphia, PA 19104-4399 USA

**Keywords:** Juvenile spondyloarthritis, Juvenile idiopathic arthritis, Enthesitis-related arthritis, Uveitis, HLA-B27

## Abstract

**Background:**

Juvenile spondyloarthritis (JSpA) represents a group of inflammatory arthritides with several distinctive features (enthesitis, involvement of spine and sacroiliac joint, HLA-B27 association and development of uveitis). There are limited data on the course of uveitis in children with JSpA.

This study aims to estimate the prevalence of uveitis and to look at the presence of HLA-B27 in relation to uveitis occurrence and ocular symptoms in a cohort of JSpA patients.

**Findings:**

This is a cross sectional/retrospective study involving patients with JSpA followed in a tertiary referral hospital.

Two hundred twenty-three patients were enrolled in the study. The prevalent diagnosis was enthesitis-related arthritis (ERA) (62%) followed by juvenile psoriatic arthritis (PsA), undifferentiated arthritis (UA), and the arthropathies associated with inflammatory bowel disease (IBD-A) (18, 14, 6%, respectively).

Uveitis was reported in twenty-four patients (11%) of the JSpA cohort (JSpA-U). ERA patients had the highest uveitis prevalence (ERA-U) (13%) with similar prevalences in UA, PsA and in IBD-A (7% each).

The prevalence of HLA-B27 positivity was similar amongst the entire JSpA-U cohort (*N* = 22, 45%) and those with ERA-U (*N* = 8, 44%). The overall prevalence of symptomatic uveitis was 79%. Neither the likelihood of uveitis, nor of symptomatic uveitis, varied by HLA-B27 status either in the entire cohort nor in those with ERA.

**Conclusions:**

About one-tenth of patients developed uveitis, the majority of which was symptomatic. Fewer than half of the patients with uveitis were HLA-B27 positive. HLA-B27 status was not statistically associated with either the development of uveitis or symptomaticity of uveitis.

## Introduction

Spondyloarthritis (SpA) represents a group of inflammatory arthritides affecting both adults and children with several distinctive features such as the presence of enthesitis, the potential involvement of spine and sacroiliac joint, a strong association with HLA-B27 and development of uveitis that is more often acute onset and/or symptomatic [1].

International League of Associations for Rheumatology (ILAR) classification of juvenile idiopathic arthritis (JIA) [2] does not recognize a specific category for Juvenile SpA (JSpA) patients. Yet, spondyloarthritis includes enthesitis-related arthritis (ERA), juvenile psoriatic arthritis (PsA), undifferentiated arthritis (UA), reactive arthritis (ReA), and the arthropathies associated with inflammatory bowel disease (IBD-A) [1]. Adult SpA has been extensively studied and similar manifestations are assumed to be true for JSpA, although they have not been verified in children.

More than one-third of adult SpA patients develop ocular inflammation that usually is characterized by acute episodes of uveitis [3, 4]. While children with JSpA are de facto expected to follow similar courses as adults, there are limited data on JSpA associated uveitis in children. In children with JSpA, uveitis is thought to be symptomatic acute anterior uveitis [5].

This study aimed to estimate the prevalence of uveitis and to look at the presence of HLA-B27 in relation to uveitis occurrence and ocular symptoms in a cohort of JSpA patients.

## Methods

This is a cross sectional/retrospective study involving patients with JSpA followed in a tertiary referral hospital. All patients evaluated by a pediatric rheumatologist at the Children’s Hospital of Philadelphia (CHOP) from February 2016 to August 2019 and diagnosed with ERA, PsA, UA or IBD-A were approached to participate in the JSpA Registry, with a 91% recruitment rate. IBD was not considered an exclusion for a JIA subtype. A RedCAP registry included responses from electronic questionnaires completed by the consenting patients and clinical data collected retrospectively from the electronic and paper medical records.

History of uveitis was established using either chart review of ophthalmology records or patient/caregiver reports; both were recorded when available. In the registry, patients were asked about ocular symptoms that occurred since the last visit. Ocular symptoms were defined as the presence of pain, redness, or photosensitivity.

Data were analyzed using Stata 15.1. Differences in categorical demographic and clinical characteristics between subcohorts were assessed using the Fisher’s exact-test. Nominal statistical significance was defined as a 2-tailed *p* value of ≤0.05. The study was reviewed and approved by the institutional review board of CHOP.

## Results

Two hundred twenty-three patients were enrolled in the study (Table 1) with 1.4 years median follow-up time (IQR 0–2.9 years). The prevalent diagnosis was ERA (62%) followed by PsA, UA and IBD-A (18, 14, 6%, respectively). Twelve ERA patients and 1 UA patient developed IBD. Amongst the entire JSpA cohort, as well as in patients with UA, sex was equally distributed (50% male). The ERA subcohort had the highest male prevalence (59%), whereas the PsA subset showed a female preponderance (20% male) (distribution of sex by group, *p* < 0.05). The rate of HLA-B27 positivity of the whole cohort was 38%; those with ERA had the highest rate of HLA-B27 positivity whereas non-ERA patients had a significantly lower rate (46% vs. 21%, *p* = 0.00). HLA-B27 data was missing from 23 of 223 JSpA patients (10%): 6 ERA, 7 PsA, 4 UA and 6 IBD.

**Table 1:** General cohort characteristics

	All JSpA (*N* = 223)	ERA (*N* = 138)	PsA (*N* = 41)	UA (*N* = 30)	IBD-A (*N* = 14)	*p*-value*
Males	111/223 (50%)	81/138 (59%)	8/41 (20%)	16/30 (53%)	6/14 (43%)	0.000
HLA-B27+ **	75/200 (38%)	61/132 (46%)	4/34 (12%)	9/26 (35%)	1/8 (12%)	0.001
Uveitis	24/223 (11%)	18/138 (13%)	3/41 (7%)	2/30 (7%)	1/8 (7%)	0.735
HLA-B27+ U †	10/22 (45%)	8/18 (44%)	0/2 (0%)	1/1 (100%)	1/1 (100%)	0.330
Symptomatic-U	19/24 (79%)	15/18 (83%)	2/3 (67%)	1/2 (50%)	1/1 (100%)	0.438

*ERA* Enthesitis-related arthritis, *PsA* psoriatic juvenile idiopathic arthritis, *UA* undifferentiated juvenile idiopathic arthritis, *IBD-A* arthropathies associated with inflammatory bowel disease, *JSpA* juvenile spondyloarthritis, *N* number of patients. *HLA-B27 + -U* HLA-B27 + -Uveitis. *Symptomatic-U* Symptomatic-Uveitis. * *p* value for Fischer’s exact test comparing distribution between JSpA subcohorts. **HLA-B27 status not available for 23 subjects – 6 ERA, 7 PsA, 4 UA, and 6 IBD-A. †Denominator is number of uveitis patients with HLA-B27 status known

Uveitis occurred in twenty-four patients (11%) in the JSpA cohort (JSpA-U) (Table 1). Patients with uveitis had a median age at diagnosis of 7.8 years (IQR 5.4–12.4 years). There was no statistically significant difference in uveitis prevalence between all JSpA subcohorts nor when ERA patients were compared to all others (*p* = 0.19, data not shown). Half of those with uveitis were male, both in the entire JSpA cohort and the ERA subcohort (46 and 50%, respectively), whereas PsA patients with uveitis were mostly female (67%). The rate of HLA-B27 positivity was 45% amongst JSpA-U (22/24 subjects with available data) and varied between subsets (*p* = 0.001). When the missing HLA-B27 from each subgroup was analyzed as all positive or all negative, the difference in HLA-B27 distribution between subgroups persisted (*p* = 0.092, *p* = 0.001) (data not shown). The overall prevalence of symptomatic uveitis was 79% and did not vary significantly between subgroups. Considering all non-ERA patients, 6 of 85 had uveitis (7% non-ERA vs 13% ERA; *p* = 0.187), which was symptomatic in 4 cases (67% non-ERA vs 83% ERA; *p* = 0.568).

We further investigated the type of “ever” ocular symptoms reported by patients. The majority of patients had both eye pain and light sensitivity, although more patients reported red eyes than of eye pain/light sensitivity (79 and 71%, respectively).

In analysis restricted to HLA-B27+ patients, the overall uveitis prevalence was 13% (the same as the ERA subset) (Table 2). The distribution of HLA-B27+ uveitis did not vary significantly in categorical subgroup comparison or in a dichotomous comparison of ERA vs. non-ERA. The difference remained not significant in a sensitivity analysis accounting for all missing HLA-B27 as positive or negative (1 PsA, 1 UA) (*p* = 0.161, *p* = 0.281, data not shown). The majority of the uveitis in these ERA patients was symptomatic (88%) (Table 2).

**Table 2:** Patients with HLA-B27 sorted by the presence of uveitis

	All JSpA (*N* = 75)	ERA (*N* = 61)	PsA (*N* = 4)	UA (*n* = 9)	IBD-A (*n* = 1)	*p*-value*	*p*-value**
Uveitis	10/75 (13%)	8/61 (13%)	0	1/9 (11%)	1/1 (100%)	0.243	1.000
Symptomatic Uveitis	8/10 (80%)	7/8 (88%)	0	0	1/1 (100%)	0.378	0.636

*ERA* Enthesitis-related arthritis, *PsA* psoriatic juvenile idiopathic arthritis, *UA* undifferentiated juvenile idiopathic arthritis, *IBD-A* arthropathies associated with inflammatory bowel disease, *JSpA* juvenile spondyloarthritis. * *p* value for the Fisher’s exact test comparing subcohorts. ** *p* value for the Fischer’s exact test ERA vs. non-ERA subsets

The presence of HLA-B27 was not statistically associated with uveitis occurrence or with the presence of ocular symptoms (Fisher’s exact test or logistic regression) either in the JSpA-U cohort as a whole or in ERA-U subset (data not shown).

## Discussion

To date, few data have been published on uveitis prevalence among children and adolescents with spondyloarthritis. This is the first study specifically focusing on JSpA related uveitis. It provides new insight on JSpA related uveitis in terms of prevalence, clinical information, and HLA-B27 status.

Current knowledge on uveitis in JSpA patients has been abstracted from adults with SpA and JIA associated uveitis. Indeed, the previous reported JSpA cohorts are small and analysis does not focus on ocular inflammation. Two recently published JSpA cohorts, one from France (114 patients) and one from Germany (118 patients) shared similar features: ERA patients as the prevalent subset (69 and 52%), male predominance (63 and 73%) and high rate of HLA-B27 positivity (43 and 66%) [6, 7]. Another study of JIA reported an HLA-B27 prevalence of 71, and 87% for ERA and UA, respectively [8]. Data on JSpA uveitis were only provided in the German cohort: the uveitis rate was 7%, without further description of associated characteristics [6].

Similar to these cohorts, the ERA subset was the most represented in our population. The high rate of PsA patients in our cohort may explain the absence of male prevalence and lower rate of HLA-B27 positivity compared to other studies [6–8]. This may also contribute to the higher rate of uveitis in our cohort.

Studies in the German and Canadian registries examined the prevalence and presentation of uveitis in different JIA subtypes [9, 10]. The uveitis prevalence in the PsA subset in our study was comparable to those in the two registries (7% in our study and 10% in both German and Canadian registries). Whereas in our study, the ERA subcohort had a higher percentage of uveitis than in the Canadian and German registries (13% vs 8 and 7%, respectively), and the UA subcohort had a higher percentage than the Canadian but not German registries (7% vs 1 and 7%, respectively) [9, 10]. Amongst ERA patients, the rate of symptomatic uveitis in our cohort was higher than in the German registry (83% vs 67%), but the rate of HLA-B27 positivity among ERA patients with uveitis was lower than in the German cohort (44% vs. 75%) [10].

Comparing our results to what is described in adult SpA, we identified a lower prevalence of uveitis. About one-third of adults with SpA develop uveitis [3, 4], whereas in our cohort, uveitis prevalence was lower. Similarly to SpA, JSpA patients were more likely to develop symptomatic rather than asymptomatic uveitis. While the prevalence of uveitis is significantly higher in HLA-B27+ adults [4], that was not the case in our cohort.

This observational study has some limitations. As patients must consent to inclusion, this study only approximates the prevalence of uveitis in JSpA patients. Another limitation is the dependence on patient recall of symptomaticity of uveitis rather than determination from chart review of individual ophthalmologic visits. Missingness of data on HLA-B27 status was another limitation, although only missing on 2 patients with uveitis. While we were unable to evaluate other risk factors (e.g. ANA status) or clinical features (uveitis location and course), this should not impact the observations we could make on uveitis prevalence in JSpA.

In conclusion, we have described characteristics of uveitis in a large cohort of JSpA patients. The prevalence of uveitis was lower than hypothesized and than has been reported in adult SpA patients. The majority reported ocular symptoms along with uveitis and HLA-B27 status was not associated with either the development, or with symptomaticity, of uveitis.

## Data Availability

The datasets used and/or analyzed during the current study are available from the corresponding author on reasonable request.

## Abbreviations

SpA: Spondyloarthritis
JSpA: Juvenile spondiloarthritis
ILAR: International League of Associations for Rheumatology
JIA: Juvenile idiopathic arthritis
HLA-B27: Human leukocyte antigen B27
ERA: Enthesitis-related arthritis
PsA: Juvenile psoriatic arthritis
UA: Undifferentiated arthritis
ReA: Reactive arthritis
IBD-A: The arthropathies associated with inflammatory bowel disease
AAU: Acute anterior uveitis
CHOP: Children’s Hospital of Philadelphia
JSpA-U: JSpA patients with uveitis
ERA-U: ERA patients with uveitis
IRQ: Interquartile range
